# Determinants of financial distress in the European air transport industry: The moderating effect of being a flag-carrier

**DOI:** 10.1371/journal.pone.0259149

**Published:** 2021-11-15

**Authors:** Yin Shi, Xiaoni Li

**Affiliations:** Faculty of Economics and Management, Universitat Rovira i Virgili, Reus, Spain; Szechenyi Istvan University: Szechenyi Istvan Egyetem, HUNGARY

## Abstract

Due to the COVID-induced global collapse in demand for air travel, the year 2020 was a catastrophic one for the aviation industry. A dramatic drop in operating revenues along with continuing fixed expenses drained the cash reserves of airlines, with consequent risks of financial distress and, potentially, even of bankruptcy. Flag-carriers are a special group in the airline business—they are considered to have privileges in terms of the support given by governments while, on the other hand, are often viewed as having low efficiency and performance. This study aims to estimate for European airlines the interaction effect of being a flag-carrier (flagship) with the relationship between leverage, liquidity, profitability, and the degree of financial distress. Findings obtained from analysing 99 European airlines over a period of ten years, indicate that the negative influence of leverage on financial stability is higher in the case of flag carriers (flagship). The impact of liquidity and profitability on financial health is more positive for flagship than for non-flagship carriers. These findings are not limited to contributing to the existing literature, but also have significant practical implications for executives, managers, and policy makers in the European air transport sector.

## 1. Introduction

COVID-19 is a highly contagious coronavirus disease and was declared a world pandemic by the WHO (World Health Organization) on March 11th, 2020. In addition to the damage, it inflicted on global health, it also caused tremendous disruption to the global economy, probably implying a long recovery period [1]. Since its highly infectious nature is a major threat human society, countries have adopted response approaches such as lockdown and travel restrictions of different degrees to prevent further contagion. The most affected industries, such as tourism and air transport, have suffered massive losses [2,3]. The International Air Transport Association [4] financial outlook for the global air transport industry, showed that airlines were expected to have lost $118 billion in 2020. Revenues are estimated to have halved, from $838 billion in 2019 to $419 billion in 2020.

Due to lockdown and travel restriction policies, travel demand decreased sharply, and many airlines had no choice but to reduce capacity, or even cease operation. According to IATA report [1], in this highly capital-intensive sector, airlines typically only have sufficient cash reserves to cover around two months of revenue loss. COVID-19 has had a greater influence on the airline industry than previous crises such as SARS in 2003, or the 9/11 terrorist attacks in 2001, because of their more limited geographical impact or duration [5,6]. According to IATA, COVID-19 resulted in the most profound de-connection of modern society since World War II and, simultaneously, put aviation into a crisis [4]. Over 40 airlines were bankrupted up to October 2020, and more carriers were expected to fail in subsequent months [7]. Large European flagship carriers such as Lufthansa, Air France-KLM, British Airways and Iberia had their operating activities significantly affected. Some of them took actions like reducing capacity and cutting schedules, while others suspended passenger flights for a period.

Given the scale of the loss of the airline business, around $173 billion was provided by governments to financially support the industry. This, however, had limited success. “*Some airlines received aid and averted bankruptcy*, *but others got no support*. *A few of the latter have ceased operating*, *and many of the remainder have severely retrenched services*” [4]. In the case of European countries, various rescue measures were employed. Some schemes were open to all airlines. For instance, the UK provided a COVID Corporate Financing Facility and Germany allowed all airlines to defer air travel taxes. In other countries, bailout aids were basically given to the national flagship carrier. Flag carrier, according to the definition given by Cambridge Dictionary, is an airline that is or was owned by a government [8]. It used to be an element of national identity and prestige [9] and it showed the national capacity to operate airline of its own. National flag carriers were initially established and owned by governments and received strong protection and support from the state. Nowadays, in this era of competition and privatization, most countries are discarding the national airline model and start to commercialize their national carriers [10]. Although most of the European flag carriers are no longer state-owned, some of them still receive more support than other non-flag carriers do. For instance, in the current COVID pandemic situation, the French and Dutch governments jointly gave €10 billion to Air France/KLM. Germany offered a €9 billion rescue deal for Lufthansa. The low-cost carrier, Ryanair, challenged the state aid given to national carriers, arguing that it was discriminatory and “*more than €30 billion in state aid had been gifted to flag carriers”* [11]. Although this legal challenge was rejected by the top EU court, it revealed that airlines of different types were challenged in different ways by the emergency.

Since the current pandemic has left global air carriers struggling for survival in the worst situation they have ever encountered, is has become extremely important to determine corporate financial distress risks. Financial distress can be interpreted as *“a condition where financial obligations cannot be met or being met with huge difficulties”* [12,13]. Financial distress prediction has been a widely researched field of study since 1960s [14,15]. It is vital for the management of a firm because it can affect creditors, auditors, stockholders, and senior management [16]. Moreover, it can provide early-warning messages on aspects that can be seen and addressed by executives and managers. To measure a firm’s financial health, key financial ratios as leverage, liquidity, and profitability are most frequently considered [12,15,17,18].

In the air transport industry, the Altman Z-score (one of the most famous bankruptcy-forecasting models [19]), has frequently been applied to evaluate the status of financial health and, hence, to forecast the possible financial distress of an airline [2,20–26]. It has been shown to have high predictive accuracy when applied to U.S. and Indian airlines. In 2020, reference [2] applied the Altman Z-score to estimate the influence of COVID-19 on the Indian air transport industry and it successfully reflected the difficult situation that the airlines were facing: the declining tendency of Z-scores was found, indicating higher probability of bankruptcy which can be caused by the sharp fall in passenger demand due to lockdown policies.

There is an extensive literature examining the key determinants of financial distress for various sectors of different countries [12,17,27–30]. Regarding the low-cost carrier business model in the U.S., reference [30] identified 5 ratios affecting financial distress. However, to the best of our knowledge, there is no similar study for the European airline industry, and especially none with a special focus on the moderator being a flag-carrier. In the current turbulent situation of the global aviation industry, this paper contributes to the literature by providing the first evidence, for the European air transport industry, of the flagship’s moderating effect on the relationship between different financial indicators and financial. The objective of the present study is, therefore, twofold: firstly, we aim to examine the key financial ratios such as leverage, liquidity, profitability to determine if they are significantly associated to the European airline’s financial distress risk. Secondly, this study seeks to explore if such influence differs when comparing flagship with non- flagship carriers.

The present paper is organized as follow: first, a literature review was conducted on related fields of research in Section 2 and research hypotheses are presented. Section 3 introduces the methodology with sample data and models. Section 4 provides results of empirical analysis and discussions and, finally, conclusions, implications, limitations are presented in Section 5.

## 2. Literature review

### 2.1 The European airline industry

Air transport was one of the fastest growing industries [31], offering more than 80 million jobs across the world and contributing 8% to gross domestic product. Being closely related to the international trading and tourism sector, airlines carried more than 30% of international trade and 60% of international tourist travel [5,6]. Airlines are easily affected by economic difficulties, natural disasters, and man-made disasters [32]. The ongoing COVID-19 pandemic caused immense disruption to the global economy [33] and resulted in a dramatic decrease in demand for air travel. At the worst point, April 2020, some 90% of all airline operations had ceased and, for international operations, this was closer to 98% [6,5]. In the year 2020, the global airline industry suffered a net loss of 118.5 billion dollars, undertaking only 16.4 million flights which is not even half that of the previous year (38.5 million flights). Correspondingly, half of the 87.7 million jobs that aviation was supporting before the crisis were at risk in 2020.

Europe is the one of the biggest aviation markets in the world. Since many border restrictions remain in place, the impact of the COVID-19 pandemic on air passenger numbers across Europe will continue to worsen. It is estimated that passenger numbers fell by around 60% in 2020, which represents about 705 million passenger journeys in Europe. Passenger demand recovery is predicted not to reach 2019 levels until 2024. Moreover, the revenues of the European airlines are expected to fall by more than the demand levels, since many are selling tickets with significantly discounted prices in order to stimulate air travel [4]. This sharp fall in demand and revenue led to many cases of airline bankruptcy in Europe.

Inspired by the Deregulation policy of the U.S., since the 1980s, the European Union has gradually promoted the liberalization of its air transport management system. The European trade agreement in 1992 gave more opportunities to enhance the competitive environment in European aviation [9]. This is supposed to be beneficial for consumers as it should stimulate lower prices and improved service, because this liberalization should make the market more competitive. Higher productivity and lowered unit cost should, therefore, be achieved [9,34]. Flag-carriers used to benefit from special advantages in the assignment of airports, slots, ground services and large subsidies to cover their losses [35]. To adapt the new market environment, some traditionally state-owned flag airlines, such as British Airways and Lufthansa (respectively privatized in 1987 and 1997), rebranded as successful private airlines, However, there were also flag-carriers, such as Alitalia (Italy) and Malev (Hungary), who found the transition difficult. Alitalia, the former Italian flag-carrier, is an example of state-managed failure. When it announced that it was on the verge of liquidation in 2008, to prevent it from being taken over by another country, its union still decided to reject the overall acquisition plan proposed by Air France-KLM. This despite it never having been profitable since 1999! Reference [36] reveals that Alitalia’s failure was mostly caused by the interference of politicians and a network configuration designed to meet political needs more than profitability. Malev’s failure, like other airlines in Greece, Poland and Portugal, shows that some European national carriers quickly turned from being shining lights to being burdens. Latterly, flying on behalf of one’s country was more important than making profits. Over the years, state funding led to inefficient airline operations. Ironically, this support made it even harder for many large European airlines to survive. Early in the end of 20^th^ century, E.U. regulators had already cancelled state subsidies for airlines, and the situation of many airlines became even worse. Reference [9,37] found that state ownership was an explanatory factor for lower performance among European airlines, and cases of bankruptcy also indicate that *“flag-carriers were not at ease behind the national flag”*. They need to face increasing competition, including those from low-cost carriers emerged and expanded from the 1990s onwards.

### 2.2 Financial distress prediction in airline industry and the Altman Z-score

Financial distress prediction is vital for policy makers, management, and investors [15,16,38,39]. In fact, over the past 50 years, it has been a field of increasing interest to researchers all around the world. Altman published a pioneering paper in which he firstly used multivariate discriminant analysis approach to develop a model that was claimed to be able to correctly predict the bankruptcy of 95% of public manufacturing firms one year prior to failure (and 72% two years prior) [15]. Later in 1983, Altman introduced a re-estimated model for private firms, called Z′-score model, in which substituted market value for the book value of equity. He also presented a four-variable Z´´-score model which excluded the sales/total assets ratio, claiming that it could predict bankruptcy for private firms as well as service firms [40]. In 2016, he applied the Z′′-score model to 31 European companies and three companies of non-European countries, and the results indicated that this model performs very satisfactorily in an international context [38]. In this study, we adopted the Altman Z′′-score model due to its specific characteristic for service industries [40] and it has been widely applied as the measurement of financial risk in the U.S. aviation industry [30,41,42].

In the financial distress prediction literature, statistical approaches and intelligent techniques are commonly applied for obtaining prediction models with higher accuracy. Several overview studies investigated and collected financial distress prediction models that used statistical and machine learning methods since the 1960s [16,39,43]. In the airline industry, reference [44] studied the application of various prediction models to U.S. air carriers. They mentioned two aviation industry specific models: the Airscore [45] and the Pilarski or P-score [46]. Airscore uses an MDA approach which is similar to the Altman Z-score and the P-score also borrows three ratios from the Altman Z-score. There are, however, some deficiencies in these two models. According to one of the pioneers of the Airscore, it can be biased toward the bigger carriers in the sample [44]. Reference [47] indicated that the P-score is correlated with the Altman Z-score when applied to the U.S. major carriers.

The Altman Z-score is frequently used in predicting financial distress and bankruptcy in the aviation industry [2,20–26]. It is demonstrated to be able to reflect different situations in different stage of economic cycle and it has successfully predicted insolvencies of U.S. air carriers [20]. The application of the Z-score model in aviation industry is not limited to the U.S. airlines, reference [26] applied it to Indian airlines, stating that satisfactory results has been obtained and that it could be recommended as a tool for predicting potential bankruptcy to Indian banks, shareholders and financial institutions. More recently, to estimate the COVID-19 influence on the Indian airline industry, reference [2] used the Altman Z-score model and found declining scores which were assumed to be consequences of lockdown policies and the sharp fall in passenger demand. Since the Altman Z-score is considered to be one of the pioneering models for financial distress prediction, it has commonly been used in studies as a method of estimating the degree of financial distress [2,12,30,48].

### 2.3 Determinants of financial distress and research hypotheses

There are many studies in the literature that examine the determinants of corporate financial distress [12,27,28,30]. In the aviation industry, reference [17] explored the relationship of controllable firm-specific variables and systematic risk in the U.S. airline industry. They found that debt leverage, profitability, firm size, growth, and safety were significant predictors of systematic risk. In order to find determinants of financial risk for low-cost air carriers, reference [30] collected financial data from 13 airlines that applied low-cost business model, using the Altman Z-score and the Springate S-score as indicators of financial failure (dependent variable). He found that firm leverage, assets structure, firm size, firm profitability, and liquidity affect the financial risk of airlines. Earlier, reference [12] used the Altman Z-score to represent a firm’s degree of financial distress in order to examine the effect of capital intensity on the relationship between leverage and financial distress in U.S. restaurant industry. In their study, leverage and capital intensity were used as the main variables and firm size, profitability, growth opportunity, holdings of liquid assets and economic conditions were used as control variables. In other risk determinants studies, a variety of financial variables were chosen and empirically studied [18,28,29,49]. In the present study, the adjusted Altman Z´´-score for service sector is chosen as the dependent variable to estimate a firm’s financial status (higher Z values indicate better financial conditions) and three financial variables are selected from different aspects that influence the estimation of financial performance of a firm: we use leverage, liquidity, profitability and firm size, firm age and flagship as control variable. Additionally, we use flagship as moderator to see the impact it may have on the financial health of airlines.

Leverage is widely used by researchers for evaluating a firm’s financial health [12,17,28–30,50]. This ratio is frequently applied for evaluating a firm’s capital structure. Previous research aimed to identify the factors that affect a firm’s decision on capital structure: fixed assets, non-debt tax shields, investment opportunities, firm size, volatility, advertising expenditures, probability of bankruptcy, profitability and uniqueness of the products [51,52]. In general, leverage tend to increase a firm’s risk [12] and high leveraged firms are believed by financial markets to be risky [50]. In the case of the airline industry, the extreme importance of tangible assets causes airline companies to have capital-intensive and high debt dependency structures [53,54]. It is normal to have a high degree of financial leverage as airlines tend to use large amounts of long-term debts to finance the purchase of assets [44] and keep technology updated [52]. This is in line with Trade-off theory [55,56], which indicates the existence of an optimal debt to equity proportion that can be determined by the trade-off between tax advantages and disadvantages. Airlines that hold more debt will have higher tax benefit advantages, and consequently by having more tangible assets, their bankruptcy costs tend to be relatively low compared with other sectors. That explains as well why, in the air transport industry, high debt ratios are common [53,57]. This high leverage leads to greater financial risk for shareholders. In this present study, leverage is adopted as an independent variable and is measured by debt ratio (total liabilities to total assets) [17,30,49], and the hypotheses established for this study are:

***H1a*** Leverage is negatively related to financial stability in European airlines.

***H1b*** Leverage has less negative impact on the financial stability of flag carriers than of non-flag carriers.

Previous studies indicate a positive relationship between liquidity and the degree of financial health. Liquidity, as a tool for analyzing a firm’s ability to pay off current debt obligations, has been commonly introduced to models in the field of financial risk determinants [17,30,58]. It is shown as a significant determinant in many studies, but some authors may disagree. [58] found an insignificant relationship between liquidity and systematic financial risk of airlines from North American, Europe, and Asia, which was consistent with the findings of [17] that liquidity was not significantly related to the systematic risk of the U.S. airlines. [30] had obtained similar findings towards this. Considering that a firm with greater liquidity tends to use less debt and bear lower risk of financial distress, we assume that liquidity is positively related to financial health as higher liquidity ratios imply lower risk [59–61]. In our study, the liquidity ratio is measured by the current ratio, which is obtained as current assets divided by current liabilities [17,30,62].

***H2a*** Liquidity is positively related to the financial stability of European airlines.

***H2b*** Liquidity has more positive impact on the financial stability of flag carriers than of non-flag carriers.

One of the frequently used metrics for assessing a firm’s ability to generate profits and values is the profitability ratio. It normally has a positive relationship with a firm’s financial health [12,17,49], as high profitability can improve the ability to reduce financial instability. Also, firms with a high profitability ratio tend to have more access to external financial resources with lower interest costs [53]. It is worth noting that reference [58] found that profitability had significant positive effect on the systematic financial risk of North American airlines, but this effect was not significant on European and Asian airlines. Therefore, we take profitability into consideration in our model in order to see its performance as a determinant on the financial stability of European airlines. In this present study, the profitability ratio is measured by the net profit margin which is the ratio of net income to operating revenues. This ratio is widely used by relevant studies [63–65] as it can indicate how much of each euro of revenues is left after all expenses [66].

***H3a*** Profitability is positively related to the financial stability of European airlines.

***H3b*** Profitability has more positive impact on the financial stability of flag carriers than of non-flag carriers.

Previous research has adopted firm size and firm age for constructing risk determinants models [12,17,27,29,30,49,67]. There is evidence that firm size and firm age is positively related to financial health since large companies possess more recourses and old companies have more experience and consequently, are more stable under the influence of economic, social, and political changes. They tend to have better abilities to deal with financial distress issues than do small or new companies do [68,69]. When an airline possesses more assets or has operated for more years, it tends to have more sales and potential profits. Although it is believed to be obvious that larger and elder firms have more solid financial health, it is not yet a conclusive result since some authors have obtained different findings [30] [70]. For instance, reference [30] found that firm size has a negative effect on financial failure score of U.S. airlines when applying Springate S-score [71] as an indicator of financial risk. Reference [70] stated that low-cost carriers may observe significantly negative influence of firm size toward financial performance. Therefore, firm size and age are applied here as control variables which are measured by the natural logarithms of total assets and years of operation.

Flag-carriers are the main national airlines of a country the name derives from the fact that, in the past, nations’ flags were painted on the aircraft in order to show the national capacity to run its own airlines. Flag carrier was considered as a symbol of the combination of entrepreneurial spirit and nationalism [10]. Historically, although flag-carriers were owned by national governments, nowadays, government ownership is increasingly rare. Till 2004, only 5 of 18 European countries were state-owned: Air Malta (Malta), Czech Airlines (Czech Republic), Malév Hungarian Airlines (Hungary), Aegean Airlines (Greece) and TAP Air Portugal (Portugal) [10]. However, four of them also experienced changes later. Malév Hungarian Airlines ceased operations in 2012 due to insolvency. TAP Air Portugal turned into semi-privatized in 2015, and Aegean Airlines and Czech Airlines became privately owned in 2014 and 2018, respectively. Even so, these formerly state-owned carriers still tend to be the biggest air carriers of a country and enjoy preferential rights in international operations and have advantages over other airlines. To the best of our knowledge, no previous study specifically analyses the financial status of European flag-carriers, and the current paper attempts to fill this gap.

***H4*:** Being a flag air carrier is positively related to the financial stability of European airlines.

## 3. Methodology

### 3.1 Data sampling

In order to examine the interaction effect of flagship on the influences of key financial distress ratios in the European airline industry, the dataset for testing and analysing covers available accounting and financial data of 99 European passenger airlines during the last ten years extracted from the Amadeus database. Amadeus is a pan-European database that is commonly used for European enterprise data searching. At the time of sampling, it contained comprehensive financial information on around 21 million public and private firms across Europe (including airlines of Russia, the Ukraine and Turkey). To obtain the sample of this study, we used industry classification code 5110 (Passenger air transport). In the sample, full-service carriers, low-cost carriers, hybrid business model, regional carriers and charter airlines are included. The time frame varied for different data availability. For instance, some airlines had data available for 2019, while others only went up to 2018. Our sample includes airline data for the last available ten years.

Several requirements were established for data sampling. First, the firm should have specific accounting data for calculating the Z′′-score as well as for the other ratios that are included in the model. Second, the firm should have available data for ten years in order to achieve more accurate results. After discarding companies not meeting these requirements, 990 observations remained for analysis. Of the airlines sampled, 21 are flagship. Although data for ten years is available for all sampled airlines, it is still an unbalanced panel dataset since the sampling period varies slightly by airline.

### 3.2 Variables and proposed model

Based on the literature in Sections 2.2 and 2.3, the Altman Z′′-score was adopted as the dependent variable measuring the degree of financial distress [2,12,15,30,38,48,72]. The Altman Z′′-score is an accounting-based bankruptcy prediction model, which is a modified version of the original Altman Z-score (1968) that was introduced by Altman in 1983. Altman et al. used this Z′′-score model on a sample of 31 European and 3 non-European companies because these companies are mostly privately held and non-manufacturing industry [38]. Model selection and application is consistent with Altman’s 1983 proposal since (unlike the original Z-score model of 1968 for public manufacturing firms), the Z′′-score is more practical when analysing airline industry because it is suitable for both public and private, manufacturing and non-manufacturing, firms. Their results suggest very satisfactory performances of Altman Z′′-score model in an international context. Since the research subjects of this study contain mostly privately traded airline companies, the Altman Z′′-score was chosen as the indicator of the degree of financial distress. According to the established intervals, a Z′′ value less than 1.1 reveals financial distress issues. Z′′ values between 1.1 and 2.6 indicate a grey zone, and firms with Z′′ value greater than 2.6 are classified as having no financial difficulties. Following Altman, it is considered that higher Z′′ values indicate financially healthier companies than those with lower Z′′ values [72]. A firm’s leverage ratio, liquidity ratio and profitability ratio are taken as independent variables. Firm size and age are used as control variable. Additionally, flagship is a dummy variable in order to examine its interaction effect. The previously mentioned variables (see Section 2.3) and the corresponding measurements, are shown as below in Table 1.

**Table 1:** Summary of variables and measurement.

	Variable name	Abbreviation	Measurement	Relevant research using corresponding variables
Dependent variable	Altman Z”-score	Z	6.56 (Working capital/Total Assets) +3.26 (Retained earnings/Total Assets) +6.72 (Earnings Before Interest and Taxation/Total Assets) +1.05 (Book Value of Equity/Book Value of Total Liabilities), and a constant coefficient of 3.25 for the firms that belong to emerging markets.	[12,17,30,67]
Main variables	Leverage ratio	LEV	Total liabilities/Total assets	[12,17]
	Liquidity ratio	LIQ	Current Assets/Current Liabilities	[17,30,62]
	Net operating margin	OM	Net income/Operating revenues	[63–66]
Control variables	Firm size	SIZE	Log of Total Assets	[27,29,30,49]
	Firm age	AGE	Log of number of years since foundation	[67]
	Flagship	FG	Flag carriers/other carriers	1 = Flag air carrier, 0 = Other air carriers

This study used panel data regression [12,30,73–75]. The model employed wasxfil

Zit = β0 + β1LEVit + β2LIQit + β3OMit + β4SIZEt + β5AGEit+β6FG it+ β7LEV*FGit +β8LIQ*FGit +β9OM*FGit +εit

where Z represents a firm’s degree of financial distress, measured by the Altman Z′′-score for service sector [38]. LEV represents leverage, measured by debt ratio: total liabilities to total assets. LIQ represents liquidity, measured by current assets divided by current liabilities. OM represents Net Operating Margin, measured by net income divided by operating revenues. SIZE represents firm size, measured by log of total assets. AGE represents firm age, measured by log of number of years since foundation. FG is a dummy which takes the value of either 1 if it is a flag-carrier, or 0 if it is non-flag carrier. In this study, we used the statistical computing package R Studio software for conducting panel data analysis.

## 4. Empirical results and discussions

### 4.1 Descriptive statistics

Table 2 presents a descriptive analysis of the variables used in the model for 99 European airlines over the 10-year period. The mean value of Z′′-score is 0.6975 with a range of -10.39 to 8.78 and standard deviation of 3.03. It should be recalled that a Z′′ value less than 1.1 indicates financial distress risk and greater than 2.6 indicates the opposite. The sampled airlines show a mean Z′′ value of 0.6975 which is much lower than 1.1. The median value 0.76 reveals that more than half of the sampled airlines are in an unfavourable financial condition according to this criterion. This finding is consistent with previous studies [22–25], that it is common for U.S. airlines to operate with low Z-scores. The leverage ratio varies from -3.19 to 4.42 with a mean of 1.2230, which implies that some of the sampled airlines have negative shareholder equity in some years. A negative debt ratio commonly indicates risk of bankruptcy since there are more liabilities than assets. Liquidity shows a mean value of 1.1366 with a range from 0.05 to 5.61, revealing a huge disparity between airlines in respect to the portion of liquid assets they possess. Regarding the operating margin, the mean value is -0.0048 with a range from -3.21 to 0.54, showing that many companies have negative EBITs in some years while others have high EBITs. The minimum age (13 years) is that of Nordwind airlines, and the maximum is for KLM (102 years). Sampled airlines has mean value of 25 years. Firm size, measured by normalization of total assets, shows a mean value of 0.0413 and a standard deviation of 0.125.

**Table 2:** Summary of descriptive analysis.

	n	Mean	median	min	max	stdev
Z-score	990	0.6975	0.76	-10.39	8.78	3.03
Leverage	990	1.2230	1.14	-3.19	4.42	1.64
Liquidity	990	1.1366	1.01	0.05	5.61	0.68
Net operating margin	990	-0.0048	0.02	-3.21	0.54	0.19
Age (Years)	990	30.3737	25	13	102	21.42
Size (Total assets €)	990	0.0413	0.001	0.000	1	0.125

Table 3 shows the matrix of Pearson’s correlation analysis. The dependent variable, Z′′-score, shows a negative correlation with leverage (r = -0.419, p <0.001), and a positive correlation with liquidity (r = 0.616, p <0.001), operating margin (r = 0.241, p<0.001), firm age (r = 0.038, p = 0.23) and firm size (r = 0.015, p = 0.67). No significant correlations were found between any of the independent variables. To check if multicollinearity existed, a Variance Inflation Factor (VIF) analysis was carried out and the results indicate that the VIF values of the variables range from 1.029 to 3.496 which are below the problematic level of 10 [4,17]. Hence, no severe multicollinearity is detected in the analysis.

**Table 3:** Summary of Pearson’s correlation.

	Z	Leverage	Liquidity	Operating Margin	Age	Size
Z	1					
Leverage	-0.419***	1				
Liquidity	0.616***	0.119***	1			
Net operating margin	0.241***	0.189***	0.032	1		
Age	0.038	0.052*	0.018	0.049	1	
Size	0.015	0.105***	-0.113***	0.069**	0.299***	1

*** p < 0.001; ** p < 0.01; * p < 0.05.

### 4.2 Empirical results

Panel regression analysis has three possible choices of regression techniques: Pooled OLS (POL), a fixed effects model (FEM), or a random effect model (REM). An F test and a Hausman test were carried out to determinate which of them was most suitable for this study. The F-test result indicates that POL is not an authentic model. The Hausman test rejects the null hypothesis, suggesting that FEM is to be preferred to REM. Nevertheless, the Least Square Dummy Variable (LSDV) technique used for FEM estimation conflicts with the established dummy variable of our model. As a result, REM was chosen for conducting the regression analysis because the purpose of this study is to understand the effects of financial distress determinants in European airline industry by analysing a sample of airlines [76].

The Breusch-Pagan test for heteroscedasticity and the Breusch-Godfrey test for serial correlation were applied to the model. The H0 hypothesis was rejected, indicating that the model has heteroscedastic and serial correlation issues. To improve the robustness of the model by reducing possible errors in estimating the significance of coefficients, we followed the approach of reference [74,75] and applied Feasible Generalized Least Square (FGLS) regressions. The FGLS regression estimation results are shown in Table 4.

**Table 4:** Summary of FGLS regression results.

Step 1. Mayor effects				Step 2. Interactions			
	Estimate coefficient β	Std. Error			Estimate coefficient β		Std. Error
**Intercept**	-4.525	***p<0.001	-0.129	**Intercept**	-3.961	***p<0.001	0.115
**LEV**	-0.677	***p<0.001	-0.011	**LEV**	-0.648	***p<0.001	0.014
**LIQ**	3.573	***p<0.001	0.019	**LIQ**	3.562	***p<0.001	0.042
**OM**	3.303	***p<0.001	0.141	**OM**	2.976	***p<0.001	0.213
**FG**	-0.257	* p = 0.046	0.079	**FG**	0.335	p = 0.101	0.205
**AGE**	0.279	p = 0.187	0.212	**AGE**	0.386	p = 0.236	0.327
**SIZE**	1.438	* p = 0.027	0.108	**SIZE**	0.939	***p<0.001	0.251
				**LEV:FG**	-0.046	* p = 0.039	0.026
				**LIQ:FG**	0.358	** p = 0.006	0.131
				**OM:FG**	10.174	***p<0.001	0.357
R^2^	0.471			R^2^	0.505		
Adj R	0.466			Adj R	0.497		
F	175.093			F	124.876		
n	990			n	990		

*** p < 0.001; ** p < 0.01; * p < 0.05.

Two stages are developed (with and without moderators) in this study in order to see more clearly the interaction effect of the model. In Step 1 we examine the mayor effects and in Step 2 we introduce the moderating term of being a flag-carrier or not. The results of the robust estimators explain significant variations in Altman Z′′-scores (adjusted R^2^ of 0.466 and 0.497, respectively).

In Step 1 (major effects model without interaction), a firm’s leverage ratio LEV shows a negative and statistically significant effect on the Z′′-score (β = -0.677, p < 0.001), which implies that when a firm’s leverage ratio increases, the Z′′-score decreases, which aggravates a firm’s financial distress [12,30]. This result confirms our H1a hypothesis as leverage is negatively related to financial stability. It should be noted that the higher the financial failure score (Altman Z′′-score) obtained, the healthier the financial status suggested for a firm, and vice versa. The other two main variables (liquidity and net profit margin) are positively related to the financial failure score, with coefficients of 3.573 and 3.303 respectively—the p values are less than 0.001, confirming H2a and H3a. Regarding the control variables, firm size shows a positive relationship with the Z′′-score, indicating that large companies tend to have lower probabilities of falling into financial distress. As for the second control variable, AGE is not statistically significant in this model. Flag-carriers are more likely to have financial distress issues than other airlines, which is negatively related to the financial failure score (β = -0.257, p <0.05). So, Hypothesis H4 is rejected.

In the model with flagship as moderator, the regression results show that control variables AGE and FG do not have any statistically significant effect. SIZE is positively related to Z′′-scores and is consistent with the results of the model without moderator. Regarding the variable LEV: FG, the results confirm that, the influence on Z′′ score is negative and statistically significant (β = -0.046; p <0.05 for flag carriers), implying that leverage has more influence on financial stability in flag carriers and H1b is therefore rejected. A higher liquidity has a positive influence on financial health degree, higher in flag carriers (β = 0.358; p <0.01 for flag carriers). This result supports H2b. We also found positive and significant influence of OM: FG on Z′′ score, stronger in the case of flag carriers (β = 10.174; p <0.001), confirming hypothesis H3b.

The obtained results confirm some of our hypotheses established for this study and reject others. A resumé of the results is shown in Table 5.

**Table 5:** Supported and rejected hypotheses.

Hypotheses	*Results*
***H1a** Leverage is negatively related to financial stability of European airlines*	Supported
***H1b** Leverage has less negative impact on financial stability of flag carriers than non-flag carriers*.	Rejected
***H2a** Liquidity is positively related to financial stability of European airlines*	Supported
***H2b** Liquidity has more positive impact on financial stability of flag carriers than non-flag carriers*.	Supported
***H3a** Profitability is positively related to financial stability of European airlines*	Supported
***H3b** Profitability has more positive impact on financial stability of flag carriers than non-flag carriers*.	Supported
***H4** Being a flag air carrier is positively related to financial stability of European airlines*	Rejected

### 4.3 Discussion

Here we consider some implications of the panel data regression results. The negative impact of leverage that we found, suggests that special attention should be paid to financial debt for controlling the financial distress risk of a company. In the aviation industry, a firm’s capital structure is particularly important. Reference [19] indicated that, when analysing problems in the aviation industry, although a high labour cost, fuel cost spikes and overcapacity are clearly important, over-leverage at both the operating and financial levels is a deeper and more fundamental factor. A high-leveraged airline will be more vulnerable and tend to struggle with high financial interest costs and economic downturns, facing the risk of bankruptcy since they might fail to meet their obligations. Successful control of financial risk is highly dependent on how financial and operating leverages are managed [17].

Liquidity could alleviate the degree of financial distress because more current assets can be used to prevent or lower the undesirable sales of essential assets when a firm enters a situation of financial distress [77]. As for net profit margin, in order to scale up, it is essential is essential for managers and owners to increase a firm’s capacity to generate profits. Besides, firms with high profitability may have a better chance of obtaining lower interest rate on debt, and consequently their financial distress risks reduce [53].

Large-sized firms tend to be more capable of absorbing the impacts of economic, social, and political change and they are more likely to become profitable due to having more resources and experience as compared to small firms. Therefore, the risk of being financially distressed reduces as firm size increases [68,69,78].

It is worth noting that, the gradual privatization of flag-carriers has resulted in hardly any still being government-owned. In 1997, when the EU introduced competition into the previously protected national airlines market in order to promote efficiency through competition [10], Europe’s previously state-backed airlines faced challenges identical those of other airlines. Although flag-carriers have passenger image advantages in that they tend to be seen as patriotic, they are still under the same pressure when operating in a deteriorating economic environment with high fuel prices. Malev from Hungary and Armavia from Armenia were flag-carriers, but they went bankrupt in 2012 and 2013, respectively. It is commonly found that, in airlines that were once state-owned, it is politically difficult to discipline managers and workers and therefore low efficiency and competitiveness arise [35]. Alitalia, Italy’s former flag carrier, had been continuously losing its competitive position since the liberalization of the European aviation market and by the year 2006, its loss had increased to 2 million euros per day [79]. Reference [36] argued that this essentially was because, as a flag-carrier, Alitalia was *“mainly used as a political tool rather than operating as a competitive firm”*. They stated that, although some former flag-carriers have been transformed into successful privately-owned airlines, there are others that found it difficult to adapt to the competition.

## 5. Conclusions and implications

The goal of this study is to evaluate the interaction effect of flagship on the impacts of leverage, liquidity, profitability on financial risk in the European airline industry. A sample of ten-year financial data of 99 European passenger air carriers was empirically analysed using a panel data regression method. We chose FGLS regression for the study, and the results show outcomes that are consistent with the theoretical expectations and the established hypotheses. Firstly, the increase of the degree of debt leverage makes European airlines more vulnerable to financial distress risk, while liquidity and profitability have positive impacts on financial health. These findings are in line with the literature [12,17,30,73,74,80]. Regarding the impacts of firm size and flagship, larger carriers are less likely to fall into financial distress and flag-carriers tend to have greater financial distress risks than non-flag carriers. This study took the further step of investigating if flagship moderates the relationship between leverage, liquidity, profitability, and Z′-score. The results indicate that the negative influence of leverage on financial stability, as measured by debt ratio, is higher in the case of flag carriers. The impact of liquidity and profitability on financial health is more positive in the case of flag carriers than the non-flag ones in the European air transport market.

On the theory front, the findings of the present study enrich the literature by examining the interaction effect of flagship on the relationship between key determinants and financial distress risk in European air transport industry and separately analysing the cases of national flag-carriers and other carriers. It reflected the pressure of those former government-owned airlines that are operating in the market full of competition after liberalization.

In practical terms, the study carries significant implications for executives, managers, and policy makers in the European aviation industry—our findings can act as a reference of financial distress management when making financial strategy decisions. As a highly capital-intensive sector, the high proportion of tangible assets in the airline sector makes the cost of financial distress somewhat lower than in other industries such as IT sector. It makes executives and managers consider the use of a high-leveraged financial policy to benefit from tax deductions, as proposed by Trade-off theory. However, more attention should be paid to any early warnings of potential financial distress of the company. We find that net profit margin has a great positive influence on the financial health of a flag-carriers. Meanwhile, it plays an important role in assessing the degree of leverage, since firms with high profitability will have a higher capacity to use debt and need more benefits from tax deductions [53].

For the aviation industry, 2020 was a catastrophic year due to the collapse in demand caused by the COVID-19 pandemic. Dramatic cuts in revenues together with high fixed expenses drained airlines’ cash reserves, resulting in financial distress risks [2]. European governments have distinctive support policies to help tide airlines over the difficulties and to avoid bankruptcy. Although countries like the UK and Germany implemented plans aimed at all airlines, other countries offered help that was limited to flag-carriers or native carriers (e.g., France and Sweden). These actions may lead to a possible regression in the liberalization process of European airline market which was undertaken to promote competitiveness and effectiveness [11,35]. This unequal support may also relieve the pressure on some flag-carriers that had been trying to adapt to a higher competition market. Policy makers should take into consideration the possibility that, in the longer term, these bailout actions might set back the European air transport market.

Although the findings of the present study provide significant insights for researchers in this field of study, some limitations should be addressed. The first limitation of this study is its limited generalizability [12], the dataset used consists only of European airlines. Consequently, the findings may not generalize to airlines operating in other regions such as the U.S. and Asia. Future studies could include global data to draw more general conclusions. The second limitation arises from the selection of dependent and independent variables. To evaluate the degree of a firm’s financial distress, there are many other available models in the existing literature, such as Springate S-score model [71] and Zmijewski model [81], that could be used as alternatives to the Altman Z-score model. In regard to the independent variables, the present model considered some specific ratios. There are, however, other ratios such as solvency and short-term debt that are commonly applied. Future research could consider adding such ratios to help in evaluating firm performance from other perspectives. Moreover, although state-owned airline is rarely to be found nowadays among the European national carriers, these national flag carriers still enjoy government aid and support, especially in a turbulent time such as COVID-19 period. Future research can analyse the political pressure and state influence on the flag carriers after receiving state aid. Furthermore, we applied flagship as the moderator and obtained significant results of interactive terms. However, state ownership, as a percentage or dummy variable, firm size, and firm age, may also be recommended as moderators to obtain results from a new perspective. Finally, it would be also interesting to explore the interactive effect of flagship or state ownership with other variable as size and operating efficiency of airlines.

## Supporting information

S1 File Data availability statement. (PDF)
