# Supplementary material for: Determinants of financial distress in the European air transport industry: The moderating effect of being a flag-carrier
Source: PLoS One. 2021 Nov 15;16(11):e0259149. doi: 10.1371/journal.pone.0259149 (PMC8592452; doi:10.1371/journal.pone.0259149)
Supplement: S1 File — (PDF) [file pone.0259149.s001.pdf]

Supporting Information

S1 File. Data availability statement.

This document is a compilation of data strategy applied in the paper. In particular, the authors outline the empirical procedure of data source, collecting and filtering to reach the final sample.

Data Sources

To operationalize our model, we used data compiled by Amadeus database by Bureau van Dijk for obtaining European airlines' financial information. Amadeus provides comprehensive information on around 21 million public and private companies across Europe, with a focus on private company information. Since not all European airlines are publicly traded, the private company information that Amadeus provide is essential for achieving the research objective we proposed.

Data Collecting

In this section we explain the procedure to figure out how to obtain data from the sources used in this study with screenshots. Primarily, we collected the dataset related to European airline companies' financial data from Amadeus database. Once opening the Amadeus main webpage, we first clicked on "Industry & activities" and then clicked "industry classifications" toolbar on the left side of screen. Form there we were able to filter down to our desired industry where selected "5110 Passenger air transport" to get the list of all airline companies among the passenger transportation service companies, including full-service carriers, low-cost carriers, hybrid business model, regional carriers and charter airlines.

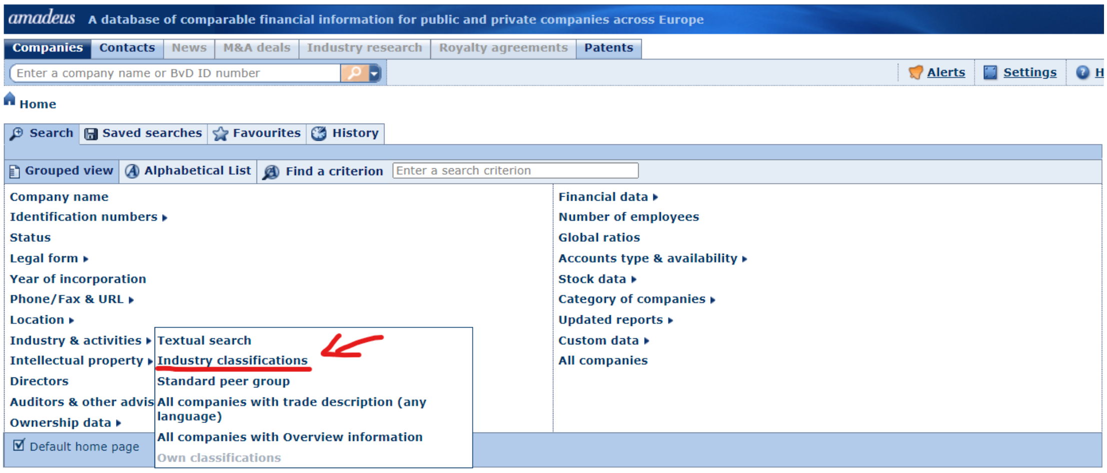

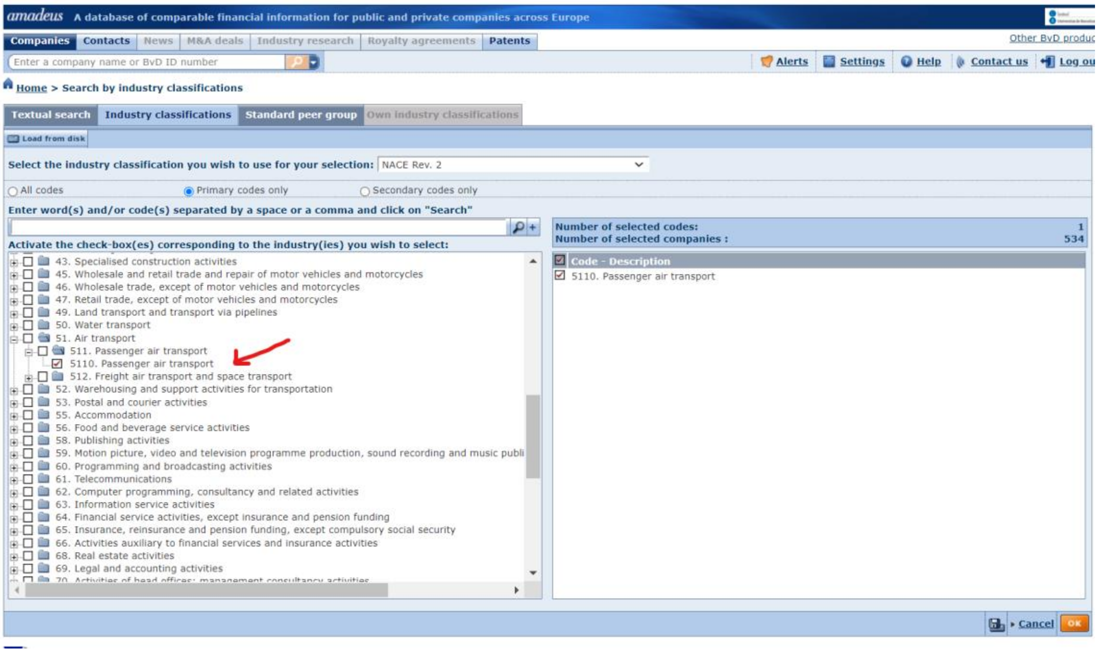

The listed airlines are automatically ordered by operating revenues of last year available from high to low. We clicked “Columns” to select financial data that are needed for our study.

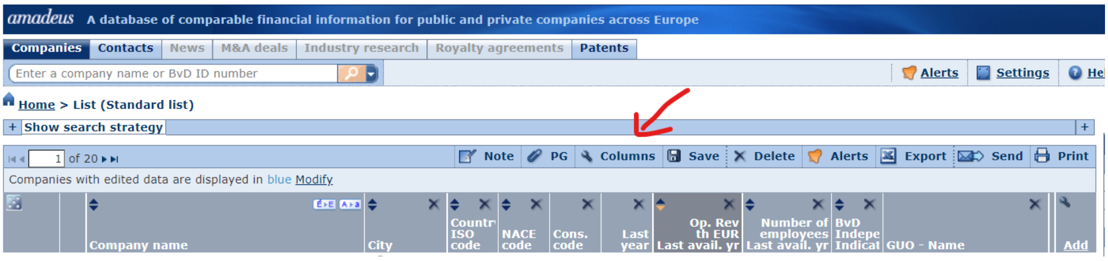

Once entered in Columns selection, we could select financial data: balance sheet and profit & lost account, and chose annual data for last ten years available, EUR in thousand, and clicked OK.

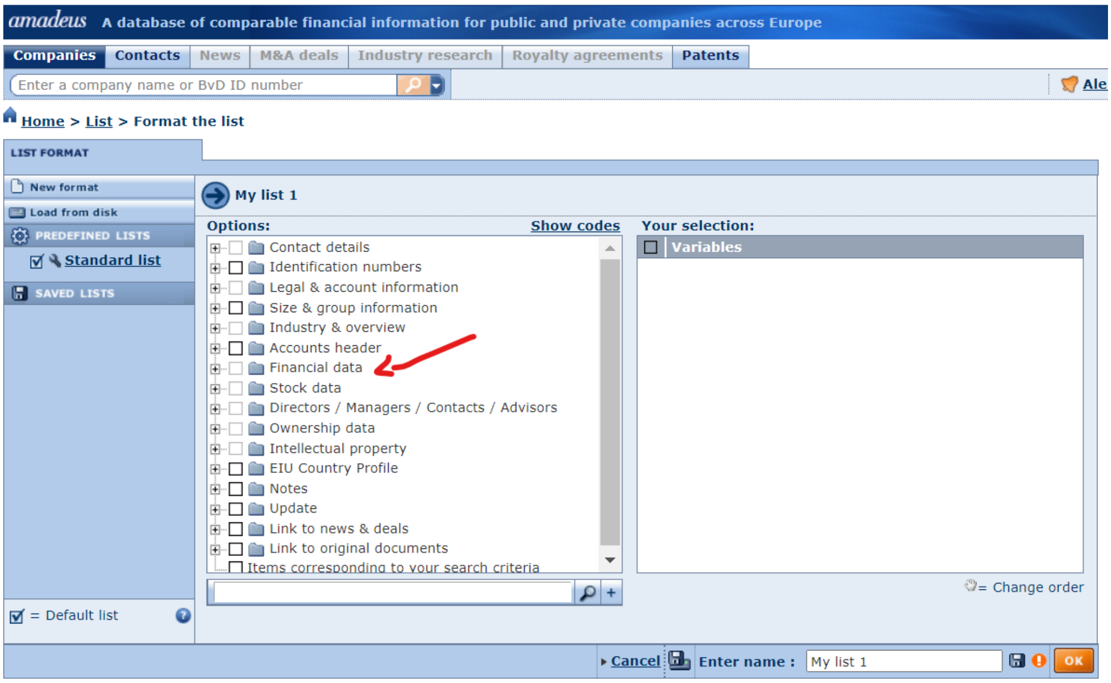

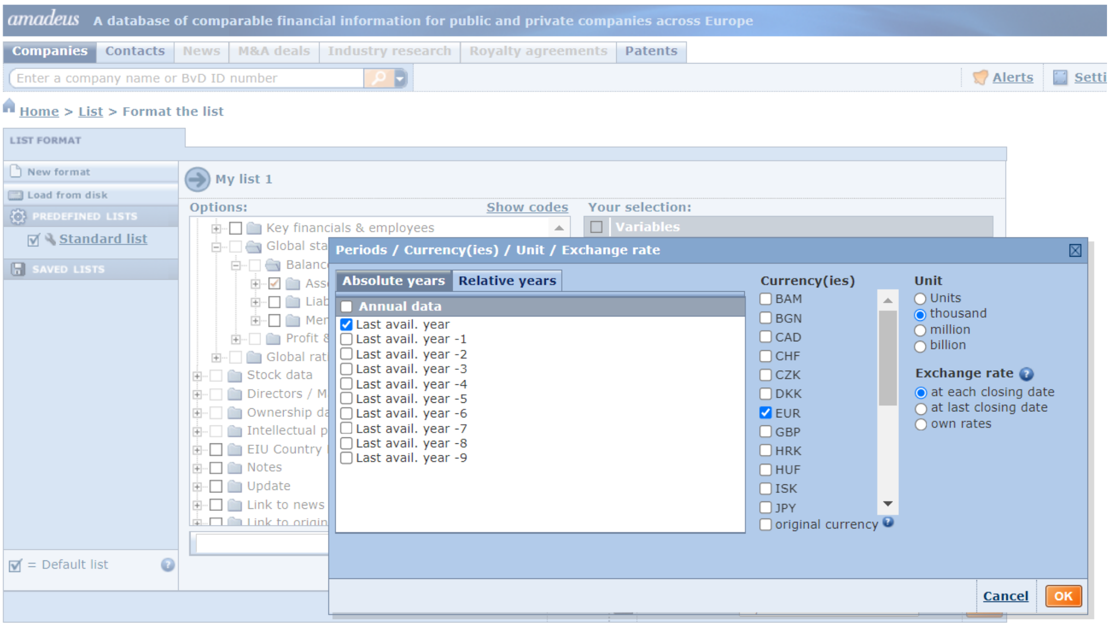

Form here we could obtain all the necessary financial data for staring to construct our dataset. We clicked Export data in excel format.

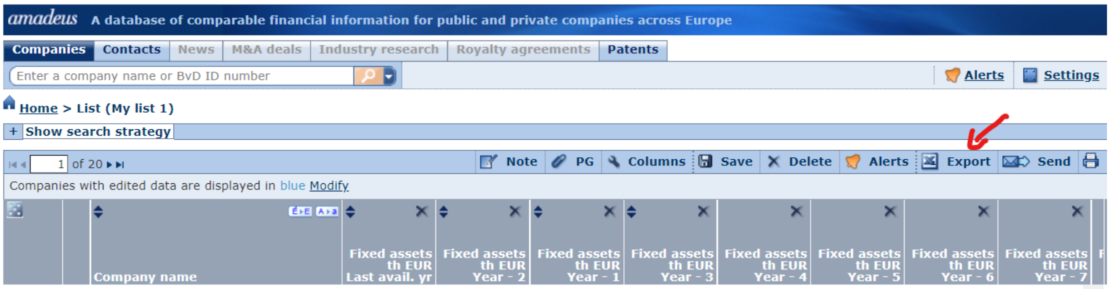

Data filtering

To construct our sample, data processing started from a set of airline companies listed in the Amadeus database over the period of last ten years. We found that not all firm-year observations were available because the Amadeus does not have all financial data for every company and some other data were unavailable. Therefore, to minimize the missing data, we select those airlines that have at least ten years of relevant accounting data which left us with 99 firms (the rest of airlines were excluded from the sample). Since the last year available data is different in each case which makes the sampling period varies slightly by airline, after removing outlier observations and filling missing values, this study obtained an unbalanced panel dataset consisted of 990 observations for the 99 sampled airlines for las ten years available.
